# Tumor Growth in the High Frequency Medulloblastoma Mouse Model Ptch1^+/−^/Tis21^KO^ Has a Specific Activation Signature of the PI3K/AKT/mTOR Pathway and Is Counteracted by the PI3K Inhibitor MEN1611

**DOI:** 10.3389/fonc.2021.692053

**Published:** 2021-07-30

**Authors:** Manuela Ceccarelli, Giorgio D’Andrea, Laura Micheli, Giulia Gentile, Sebastiano Cavallaro, Giuseppe Merlino, Giuliana Papoff, Felice Tirone

**Affiliations:** ^1^Institute of Biochemistry and Cell Biology, National Research Council (IBBC-CNR), Rome, Italy; ^2^Institute for Biomedical Research and Innovation, National Research Council (IRIB-CNR), Catania, Italy; ^3^Menarini Ricerche S.p.A., Pomezia, Italy

**Keywords:** Shh-type medulloblastoma, mouse model, PI3K/AKT/mTOR pathway, MEN1611, proliferation, apoptosis, tumor stem cells, allograft model

## Abstract

We have previously generated a mouse model (*Ptch1^+/−^/Tis21^KO^*), which displays high frequency spontaneous medulloblastoma, a pediatric tumor of the cerebellum. Early postnatal cerebellar granule cell precursors (GCPs) of this model show, in consequence of the deletion of *Tis21*, a defect of the Cxcl3-dependent migration. We asked whether this migration defect, which forces GCPs to remain in the proliferative area at the cerebellar surface, would be the only inducer of their high frequency transformation. In this report we show, by further bioinformatic analysis of our microarray data of *Ptch1^+/−^/Tis21^KO^* GCPs, that, in addition to the migration defect, they show activation of the PI3K/AKT/mTOR pathway, as the mRNA levels of several activators of this pathway (e.g., *Lars*, *Rraga*, *Dgkq*, *Pdgfd*) are up-regulated, while some inhibitors (e.g. *Smg1*) are down-regulated. No such change is observed in the *Ptch1^+/−^* or *Tis21^KO^* background alone, indicating a peculiar synergy between these two genotypes. Thus we investigated, by mRNA and protein analysis, the role of PI3K/AKT/mTOR signaling in MBs and in nodules from primary *Ptch1^+/−^/Tis21^KO^* MB allografted in the flanks of immunosuppressed mice. Activation of the PI3K/AKT/mTOR pathway is seen in full-blown *Ptch1^+/−^/Tis21^KO^* MBs, relative to *Ptch1^+/−^/Tis21^WT^* MBs. In *Ptch1^+/−^/Tis21^KO^* MBs we observe that the proliferation of neoplastic GCPs increases while apoptosis decreases, in parallel with hyper-phosphorylation of the mTOR target S6, and, to a lower extent, of AKT. In nodules derived from primary *Ptch1^+/−^/Tis21^KO^* MBs, treatment with MEN1611, a novel PI3K inhibitor, causes a dramatic reduction of tumor growth, inhibiting proliferation and, conversely, increasing apoptosis, also of tumor CD15^+^ stem cells, responsible for long-term relapses. Additionally, the phosphorylation of AKT, S6 and 4EBP1 was significantly inhibited, indicating inactivation of the PI3K/AKT/mTOR pathway. Thus, PI3K/AKT/mTOR pathway activation contributes to *Ptch1^+/−^/Tis21^KO^* MB development and to high frequency tumorigenesis, observed when the *Tis21* gene is down-regulated. MEN1611 could provide a promising therapy for MB, especially for patient with down-regulation of *Btg2* (human ortholog of the murine *Tis21* gene), which is frequently deregulated in Shh-type MBs.

## Introduction

Medulloblastoma (MB) is a highly aggressive primitive neuroectodermal tumor that originates in the cerebellum following aberrant developmental processes (1, 2). MB occurs most frequently in children (1–9 years of age); it represents 15-20% of all pediatric brain tumors and the leading cause of childhood death from cancer (3–5). Although the current treatment of disease, which includes surgery, craniospinal irradiation and high-dose chemotherapy, results in an acceptable survival rate, the prognosis of high-risk patients still remains unfavorable (4, 6, 7). Moreover, many survivors undergo serious long-term side effects, including secondary tumors (8–10), as well as physical, cognitive and behavioral impairments (11–13). Therefore, understanding the molecular mechanisms underlying MB development is now the focus of extensive research aimed to refine patient stratification and to develop personalized treatment strategies.

In this context, genomic and transcriptional profiling analyses of MBs have led to a classification of this tumor into four molecular subgroups: Wingless/Integrated (Wnt), Sonic hedgehog (Shh), Group 3 and Group 4 (14, 15). The Shh subtype comprises about 30% of all human MBs, being the most frequent in infants and adults (16, 17). The Shh-type MBs can be initiated by activation of the Shh pathway in the granule cell precursors (GCPs), located at the surface of the developing cerebellum in the external granule layer (EGL) (18–21). During cerebellar morphogenesis, the GCPs proliferate extensively in the EGL, from which they then migrate internally and differentiate into granule neurons, thus forming the internal granule layer (IGL) (22, 23). Hence, the aberrant activation of the Shh signaling pathway increases the mitotic activity of the GCPs in the EGL and promotes their transformation, leading to the development of MB (24).

Recently, we have generated a new spontaneous Shh-type MB mouse model by crossing *Patched1* heterozygous (*Ptch1^+/−^*) mice – which develop MB with an incidence of 8% to 30% depending on the genetic background (25, 26) – with mice lacking the tumor suppressor *Tis21* (27). The *Tis21* gene, whose expression levels are down-regulated in murine and human MBs (28), behaves as a MB suppressor when overexpressed in cerebellar GCPs as well as in neoplastic cells, by inhibiting cell proliferation and triggering neural differentiation (28–30). The *Ptch1^+/−^/Tis21^KO^* mice showed a significantly higher MB frequency (up to 80%) than the *Ptch1^+/−^* mice, associated to impairment of GCPs migration from the cerebellar surface to the IGL during development (27). By genomic analysis we identified the chemokine Cxcl3 as responsible for the migration of GCPs outside the EGL; in fact, in *Ptch1^+/−^/Tis21^KO^* GCPs the *Cxcl3* gene is highly down-regulated in consequence of the absence of expression of the Tis21 protein, which is recruited to the *Cxcl3* promoter and induces its activity (27). Then, we hypothesized that, in the *Ptch1^+/−^/Tis21^KO^* mouse model, the Cxcl3 down-regulation, by forcing GCPs to remain longer in the EGL under the proliferative influence of Shh instead of migrating internally and differentiating, induces the GCPs to become neoplastic cells. We confirmed our hypothesis by showing that in *Ptch1^+/−^/Tis21^KO^* mice the chronic intracerebellar administration of the chemokine Cxcl3 inhibits MB growth (31).

Moreover, a further thorough comparison between the genome-wide expression data previously obtained from GPCs at 7 day of age (P7) in the high frequency MB mouse model *Ptch1^+/−^/Tis21^KO^* and the data in the low frequency MB model *Ptch1^+/−^/Tis21^WT^* (32), indicated, as we report here, that the PI3K/AKT/mTOR pathway is up-regulated in *Ptch1^+/−^/Tis21^KO^* GCPs. The PI3K/AKT/mTOR pathway controls physiological processes such as cell growth and metabolism, proliferation, migration, survival and protein synthesis (33–35) and is frequently hyper-activated in many types of human cancer (36). In MB, the PI3K/AKT/mTOR pathway is often deregulated, contributing to the tumor development through the control of cell proliferation, chemoresistance and metastasis (37, 38). Numerous genetic alterations in the components of the PI3K/AKT/mTOR pathway have been found in human MBs, occurring independently of the molecular subtype (39, 40). In Shh-type MB, the PI3K/AKT/mTOR pathway is mutated in more than 5% of cases (41). Interestingly, activation of the PI3K/AKT/mTOR pathway is involved in resistance to Shh inhibitors (42) and in proliferation and survival of tumor stem cells (43, 44), thus contributing to the very poor prognosis of the high-risk patients.

In this report we expand the previous analyses on the *Ptch1^+/−^/Tis21^KO^* mice, to test whether the high frequency MB phenotype is caused by the enhancement of activity of the PI3K/AKT/mTOR pathway, in addition to the Cxcl3-dependent migration defect. With this aim, we first analyzed the full-blown tumors of *Ptch1^+/−^/Tis21^WT^* and *Ptch1^+/−^/Tis21^KO^* mice for mRNA and protein expression and for phosphorylation levels of the main components of the PI3K/AKT/mTOR pathway. In MB lacking the *Tis21* gene we found a hyper-activation of the PI3K/AKT/mTOR pathway, with consequent increase of cell proliferation and decrease of apoptosis. Subsequently, we analyzed the *in vivo* effect of administration of the PI3K inhibitor MEN1611 on the growth of secondary *Ptch1^+/−^/Tis21^KO^* tumors. In MB allografted mice daily treated with MEN1611 *via* oral gavage we observed a highly significant tumor growth inhibition relative to vehicle-treated mice, with a rescue of proliferative and anti-apoptotic phenotype of *Ptch1^+/−^/Tis21^KO^* MB. Notably, the inhibition of PI3K/AKT/mTOR pathway in allograft-derived nodules specifically causes the loss of tumor stem cells, strongly suggesting a role of this pathway in MB resistance and relapse. Altogether, this suggests that the high frequency MB phenotype of the *Ptch1^+/−^/Tis21^KO^* model is also caused, in cooperation with effects of the loss of *Cxcl3* expression, by activation of the PI3K/AKT/mTOR pathway.

## Materials and Methods

### Mice

The *Tis21* knockout mice were generated in the C57BL/6 (B6) strain by gene targeting of exon II of the *Tis21* gene, as previously described (45). *Patched1* heterozygous (*Ptch1^+/−^*) mice were previously created in CD1 background by replacing exons 6 and 7 of the gene with the neomycin-resistance gene cassette (26).

The crossing of *Ptch1^+/−^* with *Tis21^−/−^* mice generated *Ptch1^+/−^/Tis21^+/−^* double-heterozygous mice, which were interbred to obtain *Ptch1^+/−^/Tis21^+/+^* and *Ptch1^+/−^/Tis21^−/−^* genotypes (referred throughout this report to as *Ptch1^+/−^/Tis21^WT^* and *Ptch1^+/−^/Tis21^KO^*, respectively); the progeny was further interbred for at least six generations to achieve isogenicity. Genotyping of *Ptch1^+/−^/Tis21^WT^* and *Ptch1^+/−^/Tis21^KO^* pups was routinely performed by PCR analysis using genomic DNA extracted from tail tips, as described (27).

For *in vivo* drug studies, 4-week-old athymic nude female mice (*Foxn1^nu^/Foxn1^+^*) were purchased from Envigo (Udine, Italy) and housed in individually ventilated cages under controlled conditions (20–22°C; 55–65% relative humidity; 12/12 hours light/dark cycle; irradiated standard diet and water *ad libitum*).

All animal procedures were carried out in accordance to current guidelines of the European Ethical Committee (directive 2010/63/EU) and with the experimental protocols approved by the Italian Ministry of Health (Authorizations N. 206/2017-PR and N. 872/2015-PR). All efforts were made to minimize animal pain or discomfort.

### Microarray Analysis

Genome-wide expression study of GCPs isolated from the EGL of *Ptch1^+/+^/Tis21^WT^* (*n* = 3), *Ptch1^+/+^/Tis21^KO^* (*n* = 3), *Ptch1^+/−^/Tis21^WT^* (*n* = 4) and *Ptch1^+/−^/Tis21^KO^* (*n* = 4) mice of either sex at P7 was previously performed with Whole Mouse Genome Microarrays (Agilent Technologies, Santa Clara, CA, USA), as described (27).

Raw data from microarray experiments were processed and analyzed using GeneSpring 11.5.1 (Agilent Technologies) (27). In detail, raw signal values were thresholded to 1, log_2_ transformed, normalized to the 50^th^ percentile, and baselined to the median of all samples. Genes with a corrected *p* value of < 0.05 [one-way ANOVA followed by the Benjamini and Hochberg false discovery rate (FDR) and the Tukey’s *post hoc* test] were considered differentially expressed. The gene array expression data are shown as heat map in published reference (27) and as supplementary table of reference (27) at http://www.inmm.cnr.it/tirone/. Furthermore, the whole microarray datasets are deposited at the Gene Expression Omnibus (GEO) repository with Accession Numbers GSE178122 and GSE178124 (https://www.ncbi.nlm.nih.gov/geo/).

An enrichment analysis for Gene Ontology Biological Processes was performed in this study by Panther Classification System through The Gene Ontology Resource website^1^, using a subset of 13 deregulated genes, listed in **Figure 2**, found in literature to be involved in the PI3K/AKT/mTOR pathway. Data settings and results of the overrepresentation analysis are available in the **Table S1**.

### Generation and Collection of Tumors in *Ptch1^+/−^* Background

su *Ptch1^+/−^/Tis21^WT^* and *Ptch1^+/−^/Tis21^KO^* mice of either sex were daily monitored for tumor formation for 12 months after birth. The animals were euthanized under anesthesia when they showed physical (weight loss, head doming, hunched posture, ruffling of fur, posterior paralysis) and behavioral (preferential turning to one side, impaired balance, lethargy) signs of MB; the tumors were then isolated for subsequent experiments.

For analyses of mRNAs and proteins, the tumors were snap frozen in liquid nitrogen.

For immunofluorescence staining, MBs were fixed by immersion overnight in 4% paraformaldehyde (PFA) (wt/vol) in phosphate buffered saline (PBS), then cryoprotected in 30% sucrose in PBS and frozen at −80°C until use.

For allograft propagation, *Ptch1^+/−^/Tis21^KO^* fresh tumors were minced and then dissociated into single cells using the enzymatic dissociation system previously described (46). Briefly, the tumor fragments were incubated with collagenase type IV (500 μg/ml; Sigma-Aldrich, St. Louis, MO, USA) and hyaluronidase (500 μg/ml; Sigma-Aldrich) for 30 minutes at 37°C, then strained using a cell-strainer (40 μm; BD Biosciences, San Jose, CA, USA). A yield of 4–6 × 10^7^ cells per tumor was obtained.

### Medulloblastoma Allograft Studies

Flank tumor allografts were generated as previously described (30). Briefly, nude mice of 6–8 weeks of age were injected using aseptic technique with 3.5–4.0 million MB cells suspended in 50% matrigel (BD Biosciences) in 200 μl of PBS subcutaneously into the right flank of each mouse. Once the tumors became palpable, the mice were monitored twice a week and the size of nodules was measured by digital caliper. Each tumor volume (TV) was calculated using the ellipsoid formula: TV = (L × W^2^)/2, where L is the length of the nodule and W is the width.

When the tumors reached a volume of approximately 200 to 250 mm^3^, the mice were randomized into experimental and control groups (*n* = 5 or 6 in each group, for three independent experiments) and treatment was started. Control group mice were treated with vehicle [DMSO/Cremophor EL (50/50 vol/vol) mixed solution, diluted 10 times in a diluent solution (10% 2-hydroxypropyl-β-cyclodextrin and 10% Polyethylene Glycol 400 in distillated water); all reagents were from Sigma-Aldrich]. Experimental group mice were treated with MEN1611, kindly supplied by A. Menarini Industrie Farmaceutiche Riunite S.r.l. (Florence, Italy) [dissolved in DMSO/Cremophor EL mixed solution and 10-fold diluted in diluent solution up to a concentration of 0.65 mg/ml (6.5 mg/kg/day)] for 18 consecutive days by oral gavage.

MEN1611 (also known as CH5132799 or PA799), discovered by Chugai Pharmaceutical Co., Ltd (Tokyo, Japan) (47–49) and acquired by the Menarini Group^2^, is a potent and selective orally available PI3K inhibitor (50), currently tested in phase Ib/II clinical trial for the treatment of some solid tumors (51, 52).

Tumor volume and body weight of mice were measured three times per week. At the end of treatment, all mice were euthanized with i.p. injections of tiletamine/zolazepam (800 mg/kg) and xylazine (100 mg/kg), and tumors were collected, measured (volume and weight), photographed, and pathologically examined. Tumors were divided in two parts, one was flash frozen in liquid nitrogen for molecular analysis and the other was fixed in 4% PFA in PBS by overnight immersion for immunohistochemical analysis. For protein studies (levels and phosphorylation) by Western blotting, the tumors were harvested 4 hours after the last dose of drug (42, 48).

### RNA Extraction and Real-Time PCR

RNA isolation from MBs and nodules, cDNA synthesis and real-time PCR were performed as previously described (30, 53). Briefly, total cellular RNA was extracted from each sample with Trizol Reagent (Invitrogen, San Diego, CA, USA) following the manufacturer’s instructions, then reverse-transcribed and analyzed by real-time PCR, using SYBR Green I dye chemistry in duplicate samples and a 7900HT System (Applied Biosystems, Foster City, CA, USA). Six MBs per genotype (*Ptch1^+/−^/Tis21^WT^* or *Ptch1^+/−^/Tis21^KO^*) and six nodules per treatment (MEN1611 or vehicle) were analyzed; each experiment was conducted two or three times independently. The mRNA relative expression values were obtained by the comparative cycle threshold method (54), and were normalized to those of the *TATA-binding protein* (*TBP*) endogenous control; as control calibrator, one single MB or nodule control sample over six was randomly chosen. Specific real-time PCR primers were designed by the Beacon Designer 7.1 software (Premier Biosoft International, Palo Alto, CA, USA) or by the BLAST software^3^; their sequence is available on request.

### Western Blotting

*Ptch1^+/−^/Tis21^WT^* (*n* = 6) and *Ptch1^+/−^/Tis21^KO^* (*n* = 6) snap frozen tumor biopsies, as well as flash frozen MEN1611- and vehicle-treated nodules (*n* = 5 for each treatment), were lysed into RIPA lysis buffer [50 mM Tris–HCl (pH 7.4), SDS 0.1%, sodium deoxycholate 0.25%, 150 mM NaCl, 2 mM EDTA (pH 8.0), 10% glycerol, 1% TRITON X-100], containing 1 mM phenylmethylsulfonyl fluoride (PMSF), 10 μg/ml of leupeptin and aprotinin, 10 mM β-glycerophosphate, 4 mM NaF, 0.1 mM Na_3_VO_4_, 5 mM Na(C_3_H_7_COO), and sonicated. Proteins (40 μg per sample) were electrophoretically analyzed by sodium dodecyl sulfate (SDS)−12% polyacrylamide gel electrophoresis (PAGE) and transferred to nitrocellulose membranes (Amersham™ Protran™ 0.45 μm; #10600002; GE Healthcare Life Sciences, Little Chalfont, UK), later incubated for 2 hours in blocking buffer {TBS [10 mM Tris–HCl (pH 8.0), 150 mM NaCl]−0.05% Tween−5% powdered milk}. Lysates were immunoblotted overnight at 4°C with antibodies against phospho-AKT (Ser473; #4060), phospho-S6 (Ser235/236; #2211) and phospho-4EBP1 (Thr37/46; #2855) diluted in the same blocking buffer, followed by membrane stripping and reprobing with antibodies for AKT (#9272), S6 (#2217) and 4EBP1(#9452). The antibodies, all from Cell Signaling Technology (Danvers, MA, USA), were used at a concentration of 1:1000, except for anti-phospho-AKT used at a concentration of 1:2000. All membranes were then incubated with the antibody anti-βActin (A5441; Sigma Aldrich; 1:10000), used as loading control.

The densitometric analysis of Western blots of MBs and nodules was performed with the Scan Analysis software (Biosoft, Cambridge, UK). The protein phosphorylation ratio, expressed as mean% ± SEM of densitometric values, was obtained by averaging the percentage of protein phosphorylation in each sample relative to the corresponding total protein expression.

### Immunohistochemistry, Confocal Microscopy and Quantification of Cell Numbers

Immunohistochemical analysis of primary and secondary MB tumors, using fluorescent methods, was performed as previously described (30, 53). Briefly, all samples were embedded in Tissue-Tek OCT (Sakura Finetek, Torrance, CA, USA) and cut in serial free-floating sections of 40 μm thick on a rotary cryostat at −25°C. The sections were permeabilized with 0.3% Triton X-100 in PBS and incubated for 16 hours at 4°C with primary antibodies diluted in 3% normal donkey serum in PBS. For phospho-AKT detection, the MB slices were incubated with blocking buffer (1×PBS, 0.3% Triton X-100, 1% BSA, 5% normal donkey serum) for 1 hour at room temperature and then incubated overnight at 4°C with the primary antibody diluted in blocking buffer. Primary antibodies used were a rabbit monoclonal antibody against phospho-AKT (Ser473; Cell Signaling Technology; #4060; 1:200), a rabbit monoclonal antibody against Ki67 (LabVision Corporation, Fremont, CA; clone SP6; 1:150), a rabbit polyclonal antibody against cleaved (activated) Caspase-3 (Cell Signaling Technology; #9661; 1:100), or a mouse monoclonal antibody raised against CD15 (Santa Cruz Biotechnology, Santa Cruz, CA, USA; sc-19648; 1:100). The following day, the slides were reacted with the appropriate secondary antibodies, diluted in PBS (1:200), all from Jackson ImmunoResearch (West Grove, PA, USA): a donkey anti-rabbit Cy3-conjugated (phospho-AKT, Ki67, Caspase-3), or a donkey anti-mouse conjugated to Alexa-488 (CD15). Sections were counterstained by Hoechst 33258 (1 mg/ml in PBS; Sigma-Aldrich) to visualize the nuclei.

Digital images of the immunostained sections were collected under the same parameters with an Olympus FV1200 spectral inverted laser scanning confocal microscope and were analyzed by the IAS software (Delta Sistemi, Rome, Italy).

For quantification of phospho-AKT expression, we acquired 20 representative images from 20 sections of each tumor (*n* = 4 for each genotype), spaced with intervals of 240 μm, in order to ensure a representative sampling of the whole tumor. The MB pictures were collected with identical acquisition parameters and exposure times upon setting on negative sample, obtained by omitting the primary antibody, to uniform the background, and were analyzed with the IAS software. The phospho-AKT expression was measured as a percentage ratio of cells positive to phospho-AKT staining to the total number of cells labeled by Hoechst 33258 in each medulloblastoma image.

In both MBs and secondary tumors the numbers of proliferating (Ki67^+^), apoptotic (activated Caspase-3^+^), or CD15-positive cells were measured as a percentage ratio to the total number of cells, labeled by Hoechst 33258. The mitotic or apoptotic tumor stem cells were expressed as a percentage ratio between the number of double-marked cells (CD15^+^Ki67^+^ or CD15^+^Caspase3^+^, respectively) and the total number of CD15^+^ tumor stem cells.

In MBs, cell counts were performed on at least 40 images of randomly selected fields, collected from four different tumors for each genotype. In secondary tumors, quantification of cells labeled with a single marker (Ki67 or cleaved Caspase-3) was carried out on at least 44 randomly taken digital pictures, harvested from six different nodules for each treatment. From these same nodules, approximately 24 randomly chosen fields for each sample were collected and analyzed for proliferating and apoptotic fractions of tumor stem cells.

### Statistical Analysis

Statistical analysis of gene expression data by microarray (**Figure 2**) was performed as reported above in the dedicated paragraph, and as previously described (27).

mRNA (in **Figures 3**, **8** and **9**) expression values are shown as average ± SEM and analyzed by the Student’s *t*-test. Statistical analysis of densitometric data of protein phosphorylation (in **Figures 3** and **9**), expressed as mean% ± SEM, was also performed by Student’s *t*-test.

The immunohistochemistry data of phospho-AKT expression (shown in **Figure 4**), of proliferation and apoptosis of MB or nodule cells (shown in **Figures 5** or **7**, respectively), as well as of total, mitotic or apoptotic CD15-positive cells (shown in **Figure 8**) were presented as mean% ± SEM. All these data were at first tested for the equality of variance between groups by the Levene’s and the Bartlett’s tests; if equality was not satisfied in at least one of the two tests, the data were analyzed with a non-parametric test, namely Mann-Whitney *U*-test (i.e., for quantification of proliferating and apoptotic MB cells in **Figure 5**, of Caspase-3-positive cells of nodules in **Figure 7**, and of total and apoptotic CD15-positive tumor stem cells of nodules in **Figure 8**). Data with equality of variance (i.e., phospho-AKT expression in **Figure 4**, and mitotic cells of nodules in **Figures 7** and **8**) were analyzed using the unpaired Student’s *t*-test.

Lastly, the tumor volumes and the body weights of mice involved in *in vivo* studies with the MEN1611 inhibitor (**Figure 6**) were expressed as mean ± SEM and statistically analyzed by Student’s *t*-test; also the statistical analysis of ratio of tumor weight to body weight, expressed as mean% ± SEM, was performed by Student’s *t*-test (**Figure 6**).

The statistical analyses were performed using Microsoft Office Excel (Microsoft® Excel® for Mac 2011; version 14.7.3; Microsoft Corporation, Redmond, WA, USA) and StatView 5.0 software (SAS Institute, Cary, NC, USA) and were considered significant with *p* < 0.05.

## Results

### In the High Frequency MB Mouse Model *Ptch1^+/−^/Tis21^KO^*, the PI3K/AKT/mTOR Pathway Is Up-Regulated With a Model-Specific Signature

**Figure 1:**
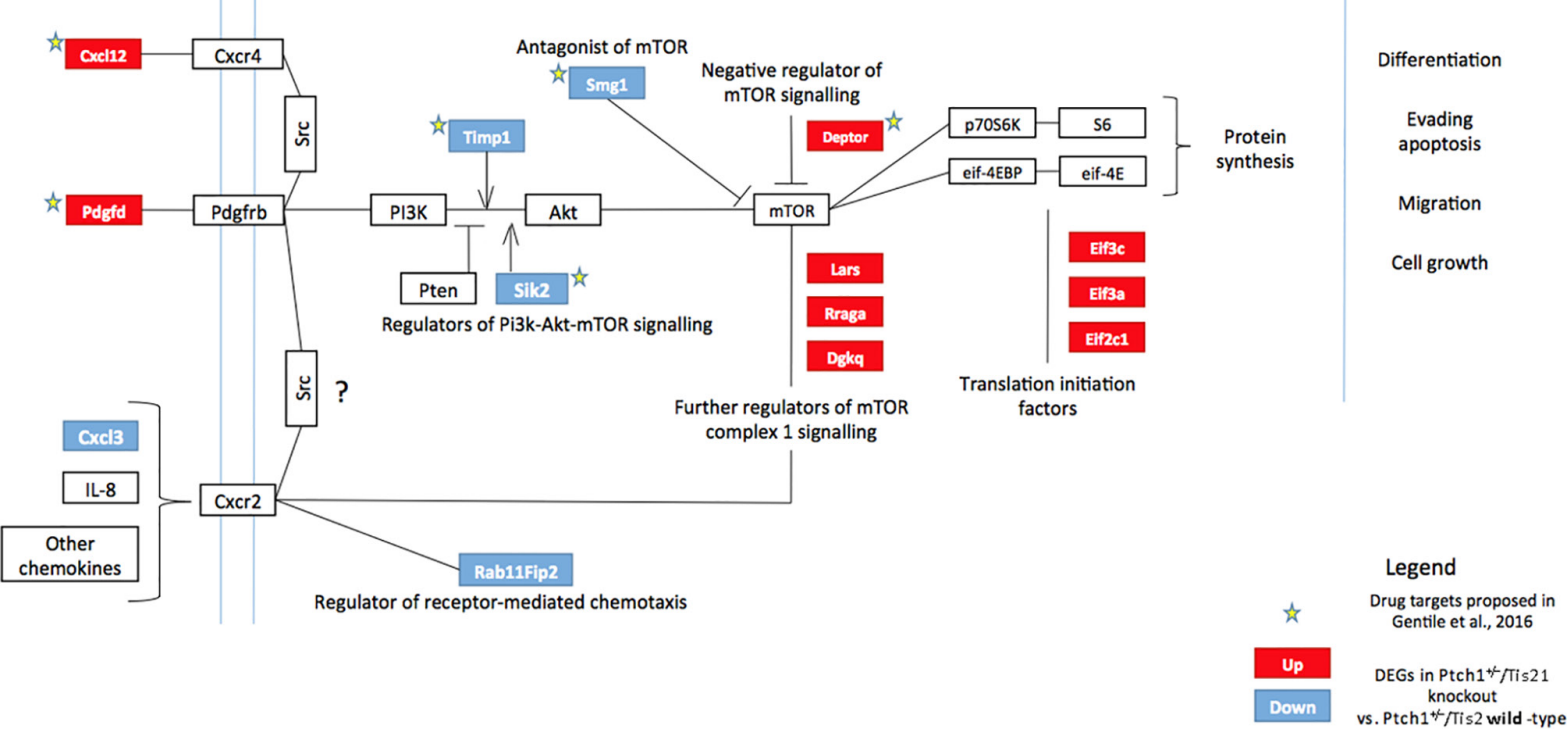
High frequency MB mouse model *Ptch1^+/−^/Tis21^KO^* has a specific activation signature of the PI3K/AKT/mTOR pathway. The picture shows the main components of the PI3K/AKT/mTOR pathway, chiefly involved in cell growth, differentiation, migration and survival. Signaling is activated at the cell membrane by different growth factors and cytokines, and is propagated through PI3K and AKT to a range of downstream molecules, including mTOR, a key regulator of protein synthesis. Several factors may constitutively activate or inhibit this signaling pathway. The colored boxes, blue or red, highlight the genes differentially expressed in the pairwise comparison *Ptch1^+/−^/Tis21^KO^* *versus* *Ptch1^+/−^/Tis21^WT^* (see **Figure 2**), identified by microarray analysis of the GCPs isolated from mice at P7, previously performed by us (27). The blue or red colors indicate down-regulated or up-regulated transcriptional expression, respectively. In *Ptch1^+/−^/Tis21^KO^* cells there is a model-specific activation of the PI3K/AKT/mTOR pathway, with extensive deregulation of the mRNA expression of upstream regulators as well as of the downstream targets. Arrows represent activation; bars represent inhibition. The stars indicate the potential drug targets, identified through the genomic analysis of deregulated *Ptch1^+/−^/Tis21^KO^*-dependent genes, as previously proposed (32). AKT, AKR mouse thymoma kinase; Cxcl, C-X-C motif chemokine ligand; Cxcr, C-X-C motif chemokine receptor; Deptor, DEP domain-containing mTOR-interacting protein; Dgkq, diacylglycerol kinase theta; Eif, eukaryotic translation initiation factor; eif-4EBP, eukaryotic translation initiation factor 4E-binding protein; IL-8, interleukin 8; Lars, leucyl-tRNA synthetase; mTOR, mammalian target of rapamycin; p70S6K, p70 ribosomal protein S6 kinase; Pdgfd, platelet-derived growth factor D; Pdgfrb, platelet-derived growth factor receptor beta; PI3K, phosphatidylinositol-3-kinase; PTEN, phosphatase and tensin homolog; Rab11Fip2, Ras-related binding protein 11 family-interacting protein 2; Rraga, Ras-related GTP-binding protein A; S6, ribosomal protein S6; Sik2, salt-inducible kinase 2; Smg1, nonsense-mediated mRNA decay-associated PI3K-related kinase; Src, steroid receptor coactivator; Timp1, tissue inhibitor of metalloproteinases-1.

Phosphatidylinositol-3-kinase (PI3K) is a heterodimeric lipid kinase, which catalyzes the generation of the second messenger phosphatidylinosityol-3,4,5-triphosphate (PIP_3_) in response to extracellular stimuli (e.g., platelet-derived growth factor, chemokines). PIP_3_ recruits many proteins to the plasma membrane, including the serine/threonine kinase AKT, which, once activated by phosphorylation, in turn phosphorylates a variety of substrates, so activating or inhibiting these targets. The positive regulation of the mammalian target of rapamycin (mTOR) activates protein synthesis and cell proliferation through the action of some translational regulatory proteins such as p70S6K and 4EBP1 (55) (**Figure 1**).

**Figure 2:**
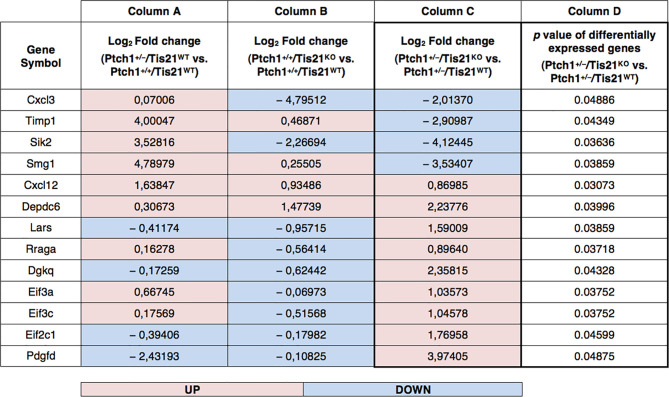
PI3K/AKT/mTOR pathway-specific differentially regulated gene expression in the *Ptch1^+/−^/Tis21^KO^* MB mouse model. As described in our previously published data, we performed a functional genomic analysis by microarray of GCPs isolated from the EGL at P7 of *Ptch1^+/+^/Tis21^WT^*, *Ptch1^+/+^/Tis21^KO^*, *Ptch1^+/−^/Tis21^WT^* and *Ptch1^+/−^/Tis21^KO^* mice (27). Through further analysis of these data, we now identify 13 genes, shown here, encoding for proteins involved in the PI3K/AKT/mTOR pathway (see **Figure 1**), whose expression significantly differs in the pairwise comparison *Ptch1^+/−^/Tis21^KO^* *versus* *Ptch1^+/−^/Tis21^WT^* (column **D**). The deregulation of these genes could explain, at least in part, the large increase in MB frequency observed in *Ptch1^+/−^/Tis21^KO^* relative to *Ptch1^+/−^/Tis21^WT^* mice. Of note, among the 13 PI3K/AKT/mTOR pathway-specific genes, the majority of them showed concordant expression changes between the pairwise comparison *Ptch1^+/+^/Tis21^KO^* vs. *Ptch1^+/+^/Tis21^WT^* (column **B**) and the pairwise comparison *Ptch1^+/−^/Tis21^WT^* vs. *Ptch1^+/+^/Tis21^WT^* (column **A**), and opposite expression changes with respect to the pairwise comparison *Ptch1^+/−^/Tis21^KO^* *versus* *Ptch1^+/−^/Tis21^WT^* (column **C**). This suggests that the activation of the PI3K/AKT/mTOR signaling is the result of a synergy between the *Ptch1^+/−^* and *Tis21^KO^* genotypes. The data in columns **(A–C)** are expressed in terms of log_2_ fold-changes of gene expression; the colors indicate overexpression (pink) or down-expression (light blue) of genes, relative to the median value. The *p* values of differentially expressed genes in the pairwise comparison *Ptch1^+/−^/Tis21^KO^* *versus* *Ptch1^+/−^/Tis21^WT^*, reported in column **(D)**, are calculated as described in the section *Microarray Analysis*.

Recently, we performed a genome-wide expression analysis by microarray of GCPs isolated from the EGL of 7-day-old (P7) *Tis21^WT^* and *Tis21^KO^* mice, either in *Patched1* wild-type background (*Ptch1^+/+^/Tis21^KO^ vs*. *Ptch1^+/+^/Tis21^WT^*) or in *Patched1* heterozygous background (*Ptch1^+/−^/Tis21^KO^* *vs*. *Ptch1^+/−^/Tis21^WT^*) (27, 32) (microarray results are available as supplementary data of reference (27) at http://www.inmm.cnr.it/tirone/ and at GEO repository, see the section *Microarray Analysis*). A further, in-depth analysis of this existing gene array expression data was performed within the set of genes significantly deregulated in *Ptch1^+/−^/Tis21^KO^* GCPs relative to the *Ptch1^+/−^/Tis21^WT^* GCPs, and focused on the PI3K/AKT/mTOR pathway, which is altered in a number of MB tumors (40). As shown here, it pointed to a subset of 13 genes belonging to this pathway and mainly to mTOR signaling, as indicated in the model of **Figure 1**. The fold changes of expression of these 13 deregulated genes are displayed in **Figure 2**. It turns out that the PI3K/AKT/mTOR pathway is up-regulated in the P7 GCPs of the high frequency MB mouse model *Ptch1^+/−^/Tis21^KO^* with a model-specific signature, since upstream activators (e.g., *Pdgfd*) as well as downstream regulators (e.g., *Lars* and *Rraga*) of this pathway are significantly up-regulated. An enrichment analysis performed by the Panther Classification system indicated that 8 over 13 genes were significantly overrepresented in the Gene Ontology (GO) Biological Process regulation of intracellular signal transduction (GO:1902531; FDR value of 0.00292; see **Table S1**). Similar results were obtained by performing an enrichment analysis for GO Biological Processes with the GSEA software^4^ v. 7.4 on the full list of genes deregulated in the pairwise comparison *Ptch1^+/−^/Tis21^KO^* vs. *Ptch1^+/−^/Tis21^WT^* (56) (data not shown). Moreover, a protein-protein analysis by the STRING software^5^ performed on the whole set of genes significantly deregulated in *Ptch1^+/−^/Tis21^KO^* GCPs relative to the *Ptch1^+/−^/Tis21^WT^* GCPs shows the interaction networks of the subset of 13 deregulated genes (**Figure S1**).

In **Figure 2**, an in-depth view of the PI3K/AKT/mTOR pathway deregulation is shown, expressed in terms of fold-changes in the comparison between *Ptch1^+/−^/Tis21^KO^* GCPs and *Ptch1^+/−^/Tis21^WT^* GCPs at P7. Of note, almost all the genes whose expression was up-regulated in this comparison, were also correspondingly down-regulated or almost unaffected in the comparison between *Ptch1^+/−^/Tis21^WT^* and *Ptch1^+/+^/Tis21^WT^* and down-regulated in the comparison between *Ptch1^+/+^/Tis21^KO^* and *Ptch1^+/+^/Tis21^WT^*, indicating that the PI3K/AKT/mTOR pathway deregulation is a synergy peculiar to the interaction between *Patched1* heterozygous and *Tis21*-null genotypes.

The down- and up-regulated genes in the *Ptch1^+/−^/Tis21^KO^ *vs. *Ptch1^+/−^/Tis21^WT^* comparison encode for proteins that control many different cellular functions and that act as activators or inhibitors of the PI3K/AKT/mTOR pathway (**Figure 1**). In addition to the *Cxcl3* chemokine, the genes down-regulated in *Ptch1^+/−^/Tis21^KO^ *GCPs include: *Timp1*, which is involved in the migration of human neural stem cells as well as leukemia cells by activating PI3K (57, 58); *Sik2*, necessary to the G1/S transition and activator of PI3K (59); and the tumor suppressor *Smg1*, which antagonizes mTOR by negatively modulating its overactivation, since *Smg1* deletion leads to hyper-proliferation (60). Conversely, in *Ptch1^+/−^/Tis21^KO^ *GCPs, the genes are up-regulated which encode for the *Cxcl12* chemokine, which stimulates PI3K/AKT/mTOR signaling activity, thereby promoting proliferation and survival of neural progenitor cells (61), and for the growth factor *Pdgfd*, which is a key activator of the PI3K/AKT/mTOR pathway in MB tumorigenesis (62, 63). Moreover, the genes up-regulated in *Ptch1^+/−^/Tis21^KO^ *GCPs include: *Depdc6* (*Deptor*), a subunit of the mTOR complex 1 (mTORC1) that can inhibit this complex and thus relieve the S6K/IRS1 feedback loop (64); the leucyl-tRNA synthetase *Lars*, that senses intracellular leucine concentration and initiates molecular events triggering mTORC1 activation, by binding Rraga GTPase (65); *Rraga*, a GTPase activator of mTORC1 (65); and *Dgkq*, which is a mediator of the mTOR complex 2 (mTORC2) (66). Finally, in *Ptch1^+/−^/Tis21^KO^ *GCPs, the genes *Eif3c*, *Eif3a* and *Eif2c1* are also up-regulated, which encode for the subunits of the mammalian translation initiation factor eIF3 (67). eIF3 functions as a hub for cellular signaling through S6K1 and mTOR/Raptor (68). Inactive eIF3 is bound to S6K1, while activated mTOR/Raptor binds to eIF3 and phosphorylates S6K1 causing its dissociation from eIF3; then, phosphorylated S6K1 phosphorylates several targets, such as eIF4B, thus implementing a translational control.

The picture of the up- and down-regulated gene expression in the comparison between *Ptch1^+/−^/Tis21^KO^* GCPs and *Ptch1^+/−^/Tis21^WT^* GCPs at P7 (**Figure 1**) indicates that several activators of the PI3K/AKT/mTOR pathway (e.g., *Lars*, *Rraga*, *Dgkq*, *Pdgfd*) are up-regulated, as well as some inhibitors (e.g., *Smg1*) are down-regulated; on the other hand, other activators of the PI3K/AKT/mTOR pathway are down-regulated (e.g., *Timp1*). Yet, the up-regulation of the translation initiation factor *eIF3* suggests that an ongoing process of activation of mTOR is occurring in *Ptch1^+/−^/Tis21^KO^* GCPs at P7, despite the fact that in these cells we do not observe an increased proliferation relative to the *Ptch1^+/−^/Tis21^WT^* GCPs (27). This could be due to the fact that, in cerebellar progenitor cells lacking *Tis21*, the control of proliferation is mainly effected by the *Tis21*-family-related gene *Btg1* as we have previously observed (69).

Therefore, in our *Ptch1^+/−^/Tis21^KO^* mouse model we see activation of the PI3K/AKT/mTOR pathway and of its upstream regulators (*Pdgfd* and *Cxcl12*) as well as downstream targets, which may also account, in conjunction with the Cxcl3-dependent impairment of migration, for the higher frequency of MB development.

**Figure 3:**
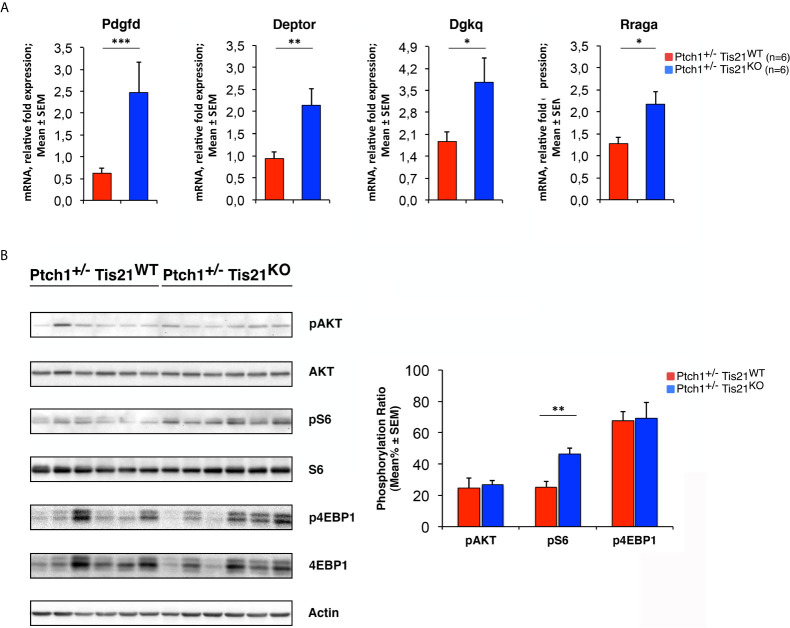
The *Ptch1^+/−^/Tis21^KO^*-specific activation signature of the PI3K/AKT/mTOR pathway is detectable also in adult MBs. **(A)** The differential expression of four representative *Ptch1^+/−^/Tis21^KO^*-specific genes, identified by microarray analysis in GCPs at P7 as indicated in **Figures 1** and **2**, was observed also in full-blown *Ptch1^+/−^/Tis21^KO^* tumors by real-time PCR, analyzing their mRNA fold expression relative to *Ptch1^+/−^/Tis21^WT^* MBs (one of them chosen for setting to unit). *TBP* was used to normalize data. Mean ± SEM fold increases are from three independent experiments; six MBs per genotype were analyzed. *p < 0.05, **p < 0.01, or ***p < 0.001, Student’s *t* test. **(B)** Protein extracts from *Ptch1^+/−^/Tis21^WT^* (*n* = 6) and *Ptch1^+/−^/Tis21^KO^* (*n* = 6) MBs were subjected to immunoblotting and densitometry analysis using antibodies against phospho-AKT (at Ser473), AKT, phospho-S6 (at Ser235/236), S6, phospho-4EBP1 (at Thr37/46), and 4EBP1. Densitometric data of protein phosphorylation, expressed as mean% ± SEM, were calculated by averaging the band intensity value of phosphorylated protein *versus* the densitometric value of total protein of each sample. **p < 0.01, Student’s *t* test.

To verify this hypothesis, we tested whether the PI3K/AKT/mTOR mRNA signature detected in P7 GCPs could be observed – by real-time PCR – also in MBs from *Ptch1^+/−^/Tis21^KO^* and *Ptch1^+/−^/Tis21^WT^* mice, and whether this corresponded with an activation of the PI3K/AKT/mTOR at the protein level. First, we found that the mRNA levels of *Pdgfd*, *Deptor*, *Dgkq* and *Rraga* genes showed a significant increase in *Ptch1^+/−^/Tis21^KO^* MBs compared with *Ptch1^+/−^/Tis21^WT^* MBs, thus matching their expression level changes observed in GCPs at P7 detected by microarray (*Ptch1^+/−^/Tis21^KO^ vs. Ptch1^+/−^/Tis21^WT^*, for *Pdgfd*: *p* < 0.001; for *Deptor*: *p* = 0.0045; for *Dgkq*: *p* = 0.0314; for *Rraga: p* = 0.0143; Student’s *t*‐test, **Figure 3A**).

**Figure 4:**
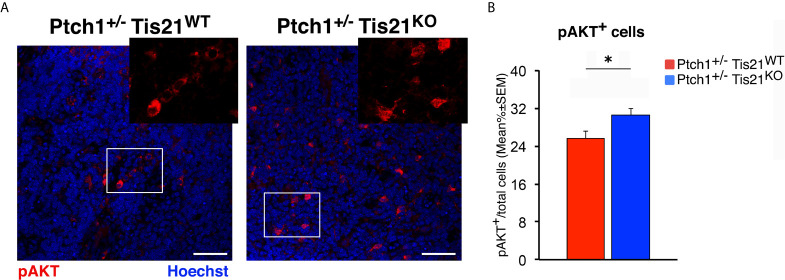
*Tis21* ablation in *Ptch1^+/−^* mice significantly increases phospho-AKT (at Ser473) expression in MBs. **(A)** Representative confocal images of the staining with the antibody against phospho-AKT (at Ser473; in red) and with the nuclear marker Hoechst 33258 (in blue) of MBs from *Ptch1^+/−^/Tis21^WT^* and *Ptch1^+/−^/Tis21^KO^* mice. Scale bar, 50 μm. White boxes, areas at higher digital magnification (2×). **(B)** The phospho-AKT expression was quantified as mean ± SEM percentage ratio of the number of phospho-AKT staining cells to the total number of cells in each field labeled by Hoechst 33258 in either *Ptch1^+/−^/Tis21^WT^* or *Ptch1^+/−^/Tis21^KO^* MBs. *p < 0.05, Student’s *t* test. MBs analyzed: *n* = 4 for each genotype; total fields analyzed: *n* = 160.

Subsequently, we investigated if these expression changes corresponded to an activation of the PI3K/AKT/mTOR pathway at the protein level. With this aim, *Ptch1^+/−^/Tis21^KO^* and *Ptch1^+/−^/Tis21^WT^* MBs were analyzed by Western blot for the phosphorylation of AKT and of downstream factors, including S6 and 4EBP1 (**Figure 3B**). In *Ptch1^+/−^/Tis21^KO^* tumor biopsy samples the mean percentage of phosphorylated AKT (at Ser473) normalized to total AKT was not significantly increased when compared with the same value found in the *Ptch1^+/−^/Tis21^WT^* MBs (*p* = 0.7774; Student’s *t*‐test, **Figure 3B**), probably because of the variability between tumors. Nevertheless, the analysis of the phosphorylation level of mTOR substrates, such as S6 at Ser235/236 and 4EBP1 at Thr37/46, revealed that the mean percentage of phosphorylated S6 protein *versus* total S6 protein presented a highly significant increase in *Ptch1^+/−^/Tis21^KO^* tumors (*Ptch1^+/−^/Tis21^KO^ vs. Ptch1^+/−^/Tis21^WT^*, for phospho-S6: 1.82-fold increase and *p* = 0.0016; for phospho-4EBP1: *p* = 0.8899; Student’s *t*‐test, **Figure 3B**). Concerning AKT, it is known that in spontaneous *Ptch1^+/−^* tumors only a low percentage of cells (about 20% of tumor mass) are positive for phosphorylated AKT (43). We reasoned that any difference of AKT phosphorylation signal might become attenuated and thus undetectable. Therefore, for a more accurate quantification we analyzed by immunohistochemistry the whole tumors section by section. We found in *Ptch1^+/−^/Tis21^KO^* MBs, a small but significant increase of the percentage of phosphorylated AKT (at Ser473), relative to *Ptch1^+/−^/Tis21^WT^* MBs (19% increase, *p* = 0.0190; Student’s *t*‐test, **Figure 4**), indicating that the PI3K/AKT/mTOR signaling is up-regulated in *Ptch1^+/−^/Tis21^KO^* MBs.

By Western blot analysis we also evaluated the activation of the MAPK pathway in tumors from *Ptch1^+/−^/Tis21^KO^* and *Ptch1^+/−^/Tis21^WT^* mice, since extensive crosstalk exists between RAS/MAPK and PI3K/AKT/mTOR signaling pathways (70) and deregulated RAS/MAPK pathway has been implicated in MB development (71). No difference was found between the two genotypes in the ERK1/2 phosphorylation ratio (data not shown), indicating that the MAPK pathway is not activated in *Ptch1^+/−^/Tis21^KO^* mice.

As a whole, the results shown above indicated that in *Ptch1^+/−^/Tis21^KO^* double-mutant mice the PI3K/AKT/mTOR pathway is up-regulated with a model-specific signature in GCPs during cerebellar development and in adult tumor cells, suggesting a pivotal role for the PI3K/AKT/mTOR pathway in the high frequency MB phenotype of the *Ptch1^+/−^/Tis21^KO^* mouse model.

### The *Ptch1^+/−^/Tis21^KO^* MBs Show Increased Proliferation and Survival of Tumor Cells

To test the effects on MB development of the up-regulation of the PI3K/AKT/mTOR pathway in *Ptch1^+/−^/Tis21^KO^* mice, we carried out an immunohistochemical analysis of neoplastic cells within the tumors obtained from *Ptch1^+/−^/Tis21^WT^* and *Ptch1^+/−^/Tis21^KO^* mice for the cellular parameters of proliferation and apoptosis.

**Figure 5:**
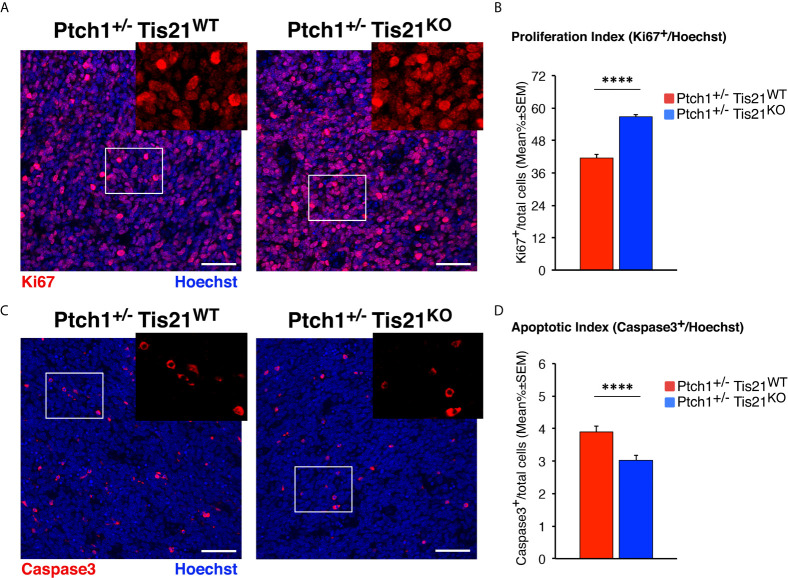
In *Ptch1^+/−^/Tis21^KO^* MBs, PI3K/AKT/mTOR activation is associated to increased proliferation and survival of tumor cells. **(A)** Representative confocal images of proliferating MB cells (labeled with the antibody against Ki67, in red) in tumors from *Ptch1^+/−^/Tis21^WT^* and *Ptch1^+/−^/Tis21^KO^* mice. Sections are counterstained with Hoechst 33258 to visualize the nuclei. Scale bar, 50 μm. The white box areas are shown with 2× digital magnification. **(B)** Quantification of mean ± SEM percentage ratio of cells positive for Ki67 to the total number of cells (Hoechst-positive; proliferation index) in MBs from *Ptch1^+/−^/Tis21^WT^* and *Ptch1^+/−^/Tis21^KO^* mice. ****p < 0.0001, Mann-Whitney *U*-test. MBs analyzed: *n* = 4 for each genotype; total fields analyzed: *n* = 320. **(C)** Representative images by confocal microscopy showing the apoptotic MB cells, identified as activated Caspase-3^+^ cells (in red), in *Ptch1^+/−^/Tis21^WT^* and *Ptch1^+/−^/Tis21^KO^* tumors. Nuclei were stained with Hoechst 33258. Scale bars: 50 μm. Cells in boxed areas are shown at higher digital magnification (2×). **(D)** Analysis in *Ptch1^+/−^/Tis21^WT^* and *Ptch1^+/−^/Tis21^KO^* MBs of the number of apoptotic cells, measured as mean ± SEM percentage ratio between number of activated Caspase-3^+^ cells and total number of cells (visualized by Hoechst 33258; apoptotic index). ****p < 0.0001, Mann-Whitney *U*-test. MBs analyzed: *n* = 4 for each genotype; total fields analyzed: *n* = 327.

To measure the proliferation rate of MB cells, tumor sections were treated with the antibody against Ki67, a protein labeling the cycling cells (72). In accordance with the activation of mTOR signaling, we observed in *Ptch1^+/−^/Tis21^KO^* MBs a highly significant increase of the proliferation index, expressed as percentage of mitotic cells to the total number of cells detected by Hoechst 33258, with respect to *Ptch1^+/−^/Tis21^WT^* MBs (36.5% increase, *p* < 0.0001; Mann-Whitney *U*-test; **Figures 5A, B**).

Afterwards, in order to measure apoptotic cells, the MB slices were reacted with the antibody for the cleaved Caspase-3, which plays a key role in programmed cell death (73). We observed that the percentage of apoptotic cells was significantly lower in *Ptch1^+/−^/Tis21^KO^* MBs than in *Ptch1^+/−^/Tis21^WT^* MBs (*p* < 0.0001 and 22% decrease; Mann-Whitney *U*-test; **Figures 5C, D**). This would agree with the observed increased phosphorylation of the S6 protein in *Ptch1^+/−^/Tis21^KO^* MBs, as S6K activity has been observed to be correlated with inhibition of apoptosis through phosphorylation of the pro-apoptotic protein BAD (74).

Several papers have shown that in MB the PI3K/AKT/mTOR pathway specifically controls proliferation and survival of tumor stem cells (43, 44, 75). Moreover, we have recently demonstrated that in *Ptch1^+/−^* mice genetic ablation of the *Tis21*-family-related gene *Btg1* increases the number of tumor cells expressing the carbohydrate antigen CD15 (53), a marker of MB stem cells (76, 77). By immunohistochemical analysis we also investigated the population of CD15^+^ cells in tumors of *Ptch1^+/−^/Tis21^KO^* and *Ptch1^+/−^/Tis21^WT^* mice, without finding any difference between the two genotypes in the total number of tumor stem cells or in their proliferating or apoptotic fractions (data not shown).

### PI3K Inhibitor MEN1611 Reduces Tumor Growth in the *Ptch1^+/−^/Tis21^KO^* MB Flank Tumor Allograft, by Inhibiting the Proliferation and Increasing the Apoptosis of Tumor Cells

We next sought to test whether the increased proliferation and decreased apoptosis observed in the *Ptch1^+/−^/Tis21^KO^* MBs, relative to *Ptch1^+/−^/Tis21^WT^* MBs, were associated with the up-regulation of the PI3K/AKT/mTOR pathway, by testing the effect of a specific inhibitor of this pathway. For this, we conducted *in vivo* studies using allograft models. *Ptch1^+/−^/Tis21^KO^* MB cells in matrigel were subcutaneously injected in the flank of athymic nude mice, then the mice were randomized into two groups and treated for 18 days with vehicle (control) or with MEN1611 at 6.5 mg/kg dose by oral gavage. MEN1611 is a new oral PI3K inhibitor targeting α, β, and γ isoforms while sparing the δ isoform of p110 catalytic subunit (50, 52), with preferential activity against the α wild-type and mutant forms, frequently involved in development and progression of various human cancers, including medulloblastoma (38, 78).

**Figure 6:**
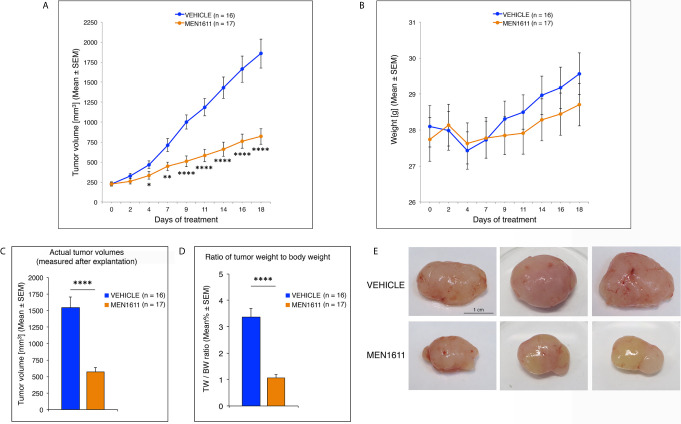
Administration of the PI3K inhibitor MEN1611 significantly reduces tumor growth in the *Ptch1^+/−^/Tis21^KO^* MB allograft. **(A)** Growth curves of nodules obtained by grafting primary *Ptch1^+/−^/Tis21^KO^* MB tumor cells subcutaneously into the right flank of nude mice. The mice were treated for 18 days with vehicle (blue) or MEN1611 (orange) at 6.5 mg/kg dose by oral gavage. The filled circles and the error bars indicate the average ± SEM of the tumor volumes measured at the days of treatment indicated. The experiment was repeated 3 times using 5–6 mice in each group (*n* = 16 for vehicle, *n* = 17 for MEN1611); there were no differences observed on anti-tumor effectiveness of MEN1611 in these three experiments. *p < 0.05, **p < 0.01, ****p < 0.0001, Student’s *t*-test. **(B)** Body weight of mice from MB allograft experiments in **(A)**. The body weight of each mouse under study was recorded on the indicated treatment days; data are expressed as mean ± SEM. The treatment with MEN1611 does not cause a significant reduction in body weight between control and treatment group, indicating the tolerability of MEN1611. **(C)** The bar graph shows the mean ± SEM of the nodule volumes measured at the end of 18 day-treatment after explantation. ****p < 0.0001, Student’s *t*-test; mice analyzed: *n* = 16 for vehicle, *n* = 17 for MEN1611. **(D)** The ratio of tumor weight (TW) to body weight (BW) was calculated for each treatment group (Vehicle: 16 mice; MEN1611: 17 mice) as mean% ± SEM. ****p < 0.0001, Student’s *t*-test. **(E)** Representative images of MEN1611- and vehicle-treated nodules are shown. Scale bar: 1 cm.

Daily oral administration of MEN1611 showed remarkable anti-tumor activity in the MB allograft model, significantly reducing the rate of tumor growth relative to vehicle-treated mice as early as the fourth day of treatment (at 4^th^ day: *p* < 0.05; at 7^th^ day: *p* < 0.01; from the 9^th^ day: *p* < 0.0001; Student’s *t*-test; **Figure 6A**). The dose of MEN1611 tested was well tolerated, as demonstrated by the absence of obvious changes in the body weight of mice (**Figure 6B**), with no toxicity or drug-related death observed in the treated animals. At the end of the 18 day-treatment, when the subcutaneous nodules were resected and measured, we verified that in the MEN1611 experimental group the tumor growth was markedly suppressed (more than 60% inhibition), because the volume of MEN1611-treated tumors was about a third of that of control nodules and, likewise, the ratio of tumor weight to body weight was significantly less in MEN1611-treated mice with respect to the vehicle-treated mice (*p* < 0.0001; Student’s *t*-test; **Figures 6C–E**).

**Figure 7:**
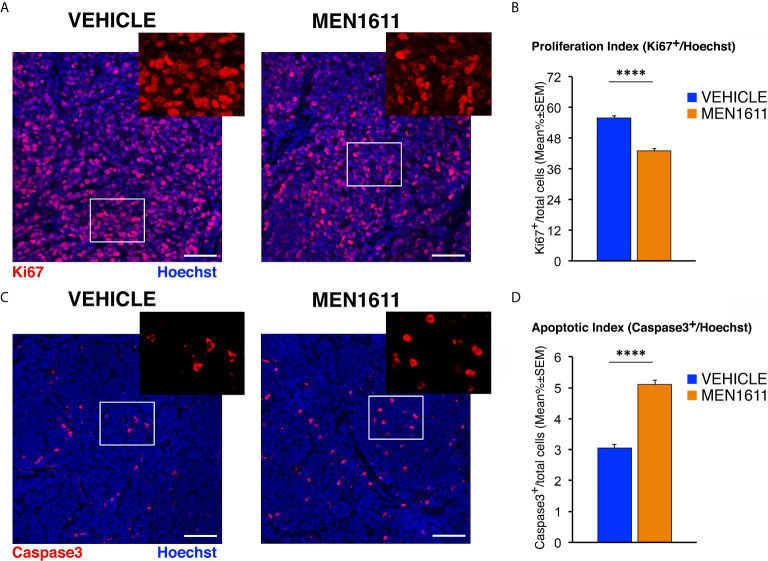
MEN1611 inhibits cell proliferation and increases apoptosis in the *Ptch1^+/−^/Tis21^KO^* secondary tumors. **(A)** Representative confocal images of mitotic cells, identified as Ki67^+^ cells (in red), in the nodules of *Ptch1^+/−^/Tis21^KO^* MB allograft treated with MEN1611 or vehicle. Sections were stained with Hoechst 33258 to visualize the nuclei. Scale bar, 50 μm. White boxes, areas at higher digital magnification (2×). **(B)** Quantification of cells positive for the proliferation marker Ki67 in the secondary tumors of mice treated with MEN1611 or vehicle, measured as mean ± SEM percentage ratio between the number of Ki67^+^ cells and the total number of cells (labeled by Hoechst 33258; proliferation index). ****p < 0.0001, Student’s *t* test. Mice analyzed: *n* = 6 for each treatment; total fields analyzed: *n* = 264 for each treatment. **(C)** Representative confocal images of nodules from MEN1611 and control groups, stained for the apoptotic marker cleaved Caspase-3 (red) and for the nuclei dye Hoechst (blue). Scale bar, 50 μm. At the top right of each tumor image, the digital enlargement (2×) of the white box area is shown. **(D)** Quantitative analysis of tumor cells expressing activated Caspase-3 in the nodules of MEN1611-treated and vehicle-treated mice. Data are mean ± SEM percentage ratio between the number of Caspase-3^+^ cells and the total number of cells, labeled by Hoechst 33258 (apoptotic index). ****p < 0.0001, Mann-Whitney *U*-test. Mice analyzed: *n* = 6 for each treatment; total fields analyzed: *n* = 287 for vehicle and *n* = 322 for MEN1611.

Next, we analyzed whether the effect of MEN1611 on the growth of secondary tumors, which histologically resembled the primary tumors from which they derived (compare **Figure 5** and **Figure 7**), is the result of inhibiting proliferation and/or increasing apoptosis of tumor cells. In MEN1611-treated nodules we observed a highly significant decrease in the percentage of Ki67^+^ cells to the total number of cells (detected by Hoechst 33258), with respect to the vehicle-treated tumors (*p* < 0.0001 and 23% decrease; Student’s *t*-test; **Figures 7A, B**). At the same time, the percentage of Caspase-3-positive cells to the total number of cells (apoptotic index) was significantly higher in MEN1611-treated than in vehicle-treated tumors (68% increase, *p* < 0.0001; Mann-Whitney *U*-test; **Figures 7C, D**).

**Figure 8:**
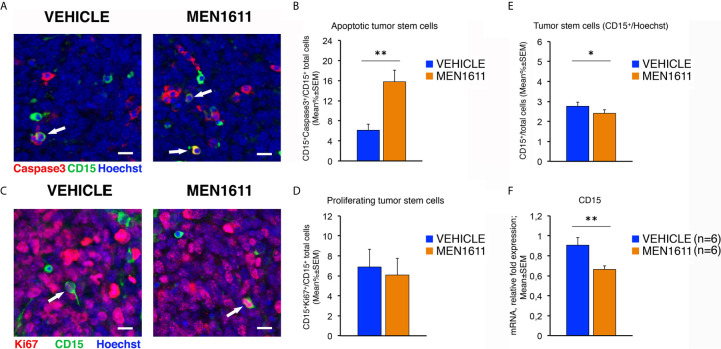
In *Ptch1^+/−^/Tis21^KO^* MB allografts, the treatment with MEN1611 induces the apoptosis of tumor stem cells. **(A)** Representative confocal images of secondary tumors from *Ptch1^+/−^/Tis21^KO^* MB allograft treated with MEN1611 or vehicle, labeled with cleaved Caspase-3 (red) and CD15 (green) markers, and the nuclei dye Hoechst (blue). The apoptotic tumor stem cells (CD15^+^Caspase3^+^) are indicated by white arrows. Scale bar, 10 μm. **(B)** Apoptotic tumor stem cells in the nodules of MEN1611-treated and vehicle-treated mice were quantified as mean ± SEM percentage ratio between the number of CD15^+^Caspase3^+^ cells and the total number of CD15^+^ cells. **p < 0.01, Mann-Whitney *U*-test. Mice analyzed: *n* = 6 for each treatment; fields analyzed: *n* = 144 for vehicle and *n* = 145 for MEN1611. **(C)** Representative confocal images of staining of MEN1611-treated and vehicle-treated nodules for Ki67 (red) and CD15 (green) markers. Nuclei were stained with Hoechst 33258. The proliferating tumor stem cells (CD15^+^Ki67^+^) are indicated by white arrows. Scale bars: 10 μm. **(D)** Quantification of the percentage ratio (mean ± SEM) of CD15^+^Ki67^+^ cells to the total number of tumor stem cells (CD15^+^) in nodules of MB allograft treated with MEN1611 or vehicle. *p* = 0.7346, Student’s *t* test. Mice analyzed: *n* = 6 for each treatment; fields analyzed: *n* = 134 for vehicle and *n* = 146 for MEN1611. **(E)** Quantification of the tumor stem cells, identified as CD15^+^ (green), in nodules of mice treated with MEN1611 or vehicle, measured as mean ± SEM percentage ratio between the number of CD15^+^ cells and the total number of cells (Hoechst-positive). The CD15^+^ cells were counted both in tumor slices reacted with the antibodies against Caspase-3 and CD15 **(A)**, and in sections labeled with Ki67 and CD15 markers **(C)**. *p < 0.05, Mann-Whitney *U*-test. Mice analyzed: *n* = 6 for each treatment; fields analyzed: *n* = 278 for vehicle and *n* = 291 for MEN1611. **(F)** Real-time PCR analysis of *CD15* mRNA obtained from MEN1611- and vehicle-treated nodules. Mean ± SEM values are from two independent experiments and are shown as fold change relative to the vehicle-treated nodules (one of them chosen for setting to unit). *TBP* mRNA was used to normalize data. Six secondary tumors per treatment were analyzed. **p < 0.01, Student’s *t* test.

Interestingly, immunohistochemical analysis of the population of tumor stem cells, labeled by their expression of the CD15 antigen, showed that in MEN1611-treated group the proportion of apoptotic cells (measured as percentage ratio between CD15^+^Caspase3^+^ double-marked cells and total number of CD15^+^ cells) was 2.5-fold increased relative to control-treated tumors (*p* = 0.0019; Mann-Whitney *U*-test; **Figures 8A, B**). Conversely, no differences were detected between MEN1611- and vehicle-treated nodules in the proliferative rate of tumor stem cells, measured as percentage ratio between CD15^+^Ki67^+^ cells and total number of CD15^+^ cells (*p* = 0.7346; Student’s *t*-test; **Figures 8C, D**). This indicates that the treatment with the PI3K inhibitor MEN1611 can specifically deplete the CD15^+^ cells, with a possibly positive impact on prognosis expectance in relapses. In fact, in MEN1611-treated nodules the number of total CD15^+^ cells was reduced slightly although significantly, with respect to the control group (*p* = 0.0263; Mann-Whitney *U*-test; **Figure 8E**). We observed in MEN1611-treated nodules a significant decrease of CD15 expression also of the mRNA levels, relative to control-treated nodules (*p* = 0.0056; Student’s *t*-test; **Figure 8F**).

Together, these results confirm the involvement of the PI3K/AKT/mTOR pathway in the phenotype of *Ptch1^+/−^/Tis21^KO^* high frequency MB model and indicate that MEN1611 can function as an anti-proliferative and a pro-apoptotic agent in the *in vivo* treatment of MB.

### In *Ptch1^+/−^/Tis21^KO^* MB Allograft, MEN1611 Inhibits PI3K/AKT/mTOR Pathway by Down-Regulating Model-Specific Genes

Next, we sought to further investigate the reduced growth of secondary tumors observed in the MB allograft by the PI3K inhibitor MEN1611, studying how in our model the enhancement of PI3K/AKT/mTOR signaling cascade observed in *Ptch1^+/−^/Tis21^KO^* MB was counteracted. For this purpose, the secondary tumors were analyzed by Western blotting for the levels of phosphorylated and total forms of various effectors of the PI3K/AKT/mTOR pathway, including AKT, S6 and 4EBP1.

**Figure 9:**
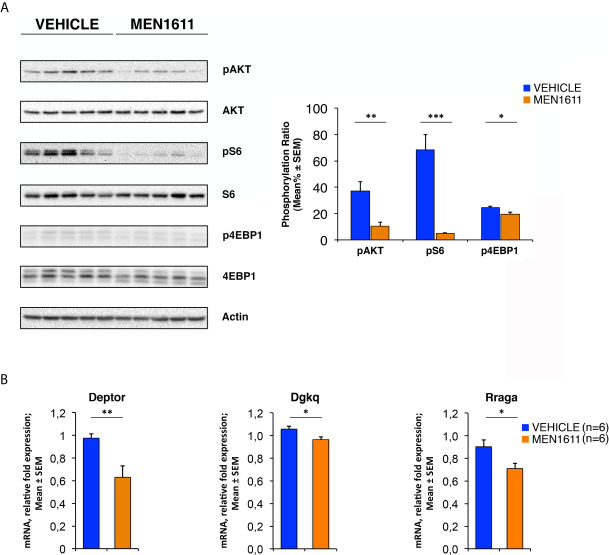
In *Ptch1^+/−^/Tis21^KO^* MB allografts, MEN1611 inhibits the PI3K/AKT/mTOR pathway by down-regulating model-specific genes. **(A)** Immunoblotting and densitometry analysis with anti-phospho-AKT (at Ser473), anti-AKT, anti-phospho-S6 (at Ser235/236), anti-S6, anti-phospho-4EBP1 (at Thr37/46), and anti-4EBP1 antibodies in MEN1611- and vehicle-treated nodules. Five tumors per treatment were analyzed. The protein phosphorylation ratio (see graph) was obtained by averaging (mean% ± SEM) the band densitometric intensity value of phosphorylated protein *versus* the densitometric value of total protein of each sample. *p < 0.05, **p < 0.01 or ***p < 0.001, Student’s *t* test. **(B)** Real-time PCR analysis of mRNA obtained from MEN1611- and vehicle-treated nodules. Six tumors per treatment were analyzed. Average ± SEM values are from two independent experiments and are shown as fold change relative to the control sample (vehicle-treated nodules; one of them was chosen for set to unit). *TBP* mRNA was used to normalize data. *p < 0.05 or **p < 0.01, Student’s *t* test.

As shown in **Figure 9A**, the allograft tumors treated with MEN1611 have an inhibited PI3K/AKT/mTOR signaling axis compared to vehicle-treated tumors, showing substantial decrease in levels of phospho-AKT, phospho-S6 and phospho-4EBP1 relative to their corresponding total forms. The densitometric analysis revealed a significantly lower level of phosphorylation of AKT at Ser473 in MEN1611-treated nodules with respect to vehicle-treated nodules (*p* = 0.0077; Student’s *t*-test; **Figure 9A**). As for the mTOR substrates S6 and 4EBP1, in the MEN1611 group we observed a significant decrease of their phosphorylation relative to the control group, with phospho-S6 showing higher statistical significance (MEN1611 *vs*. vehicle, for phospho-S6: *p* < 0.001; for phospho-4EBP1: *p* = 0.0448; Student’s *t*-test; **Figure 9A**). Interestingly, the inhibition of the PI3K/AKT/mTOR pathway by MEN1611 does not induce in the nodules the activation of the pro-survival MAPK signaling pathway (data not shown), event that often promotes resistance to single drugs (79).

Furthermore, the expression of model-specific genes related to activation of the PI3K/AKT/mTOR pathway in *Ptch1^+/−^/Tis21^KO^* mice was also down-regulated in nodules isolated from MEN1611-treated mice as compared to vehicle-treated controls. In fact, in the MEN1611 group the mRNA levels of *Deptor*, *Dgkq* and *Rraga* genes, determined by real-time PCR, were significantly decreased with respect to the control group (MEN1611 *vs*. vehicle, for *Deptor*: *p* = 0.0024; for *Dgkq*: *p* = 0.0176; for *Rraga*: *p* = 0.0207; Student’s *t*‐test, **Figure 9B**).

Overall, our results demonstrate that in MB cells the PI3K inhibitor MEN1611 rescues the *Ptch1^+/−^/Tis21^KO^*-dependent activation of the PI3K/AKT/mTOR pathway, in parallel with a decrease of tumor growth and increased death of MB stem cells, and point to treatment with MEN1611 as a promising approach for cancer therapy of MBs harboring the *Tis21* ablation.

## Discussion

We have previously generated a high frequency MB model *Ptch1^+/−^/Tis21^KO^*. The heterozygosity of *Patched1* causes constitutive activation of the Shh pathway, which is by itself a cause of tumorigenesis in GCPs, while *Patched1* homozygosity is lethal (embryos die at E9 due to failure of the closure of the neural folds) (26). In this report we show a PI3K/AKT/mTOR pathway signature identified in data belonging to a previous microarray analysis, specific for our MB mouse model. This signature denotes activation of this pathway and appears to be specifically associated to the synergic interaction between the *Tis21* knockout and the *Patched1* heterozygosity in P7 GCPs. Remarkably, this activation of the PI3K/AKT/mTOR pathway is seen not only in P7 GCPs of *Ptch1^+/−^/Tis21^KO^* mice relative to *Ptch1^+/−^/Tis21^WT^* mice, but also in *Ptch1^+/−^/Tis21^KO^* tumors relative to *Ptch1^+/−^/Tis21^WT^* tumors, where the PI3K inhibitor MEN1611 is able to counteract the ongoing tumorigenesis.

### Dual Effect of Tis21 Deletion on Ptch1-Dependent Medulloblastoma Development Through Cxcl3-Dependent Control of Migration and PI3K/AKT/mTOR Control of Mitosis/Apoptosis

The increase of proliferation observed in *Ptch1^+/−^/Tis21^KO^ vs. Ptch1^+/−^/Tis21^WT^* tumors is not present in either GCPs or MB lesions of 2-, 6- or 12-week-old mice (27), indicating that this proliferative phenotype is tumor-specific; conversely, in *Ptch1^+/−^/Tis21^KO^* cerebella during either the physiological or preneoplastic development the arrest of migration of GCPs prevails (27). However, the activation of the PI3K/AKT/mTOR mRNAs observed in P7 GCPs of *Ptch1^+/−^/Tis21^KO^ vs. Ptch1^+/−^/Tis21^WT^* mice, is propaedeutic to activation of cell division (e.g., through up-regulation of the translation initiation factors *Eif2c1*, *Eif3a*, *Eif3c*), and might be an early developmental effect that in fully developed tumors triggers increased cell division. We hypothesized that the defect of migration of the GCPs from the EGL, forcing the GCPs to remain exposed longer to the effect of Shh, may favor mutations, followed by transformation from a preneoplastic to a neoplastic state with proliferative activation. This hypothesis is supported by experimental data showing that the rescue of the defect of migration in *Ptch1^+/−^/Tis21^KO^* cerebella by exogenous administration of Cxcl3 significantly reduces MB frequency (31). Additionally, we propose here that the neoplastic transformation in the high frequency Shh-MB model *Ptch1^+/−^/Tis21^KO^* could be the result of a synergy between the *Tis21^KO^*-dependent down-regulation of *Cxcl3*, which forces the GCPs to remain in the EGL, and the activation of the PI3K/AKT/mTOR pathway, that fully displays its pro-proliferative effects when the transition from preneoplastic to neoplastic GCPs takes place. Our hypothesis is in agreement with the concept that in the MB pathology the PI3K/AKT/mTOR pathway is mostly involved in tumor promotion rather than initiation (40). Moreover, in the high frequency medulloblastoma model, the activation of PI3K pathway does not seem to be correlated to the defect of migration. In fact, in the *Ptch1^+/+^/Tis21^KO^* GCPs the defect of migration is present, relative to *Ptch1^+/+^/Tis21^WT^* GCPs (27), but there is no up-regulation of the PI3K/AKT/mTOR pathway, unlike in *Ptch1^+/−^/Tis21^KO^* (compare column B *vs*. column C in **Figure 2**).

### Different Mode of Activation of the PI3K/AKT/mTOR Pathway in Ptch1^+/−^/Tis21^KO^ GCPs, in MB and in Nodules

It is known that in different human cancers *Btg2* (human ortholog of the mouse gene *Tis21*) inhibits the processes of cell proliferation, survival and metastasis by suppressing the PI3K/AKT/mTOR pathway, since the Btg2 protein acts as a negative regulator of AKT in both tumor cells (80–82) and normal ones (83, 84). In the high frequency MB mouse model *Ptch1^+/−^/Tis21^KO^* we observed increased phosphorylation of AKT and, to a higher extent, of S6, a downstream target of mTOR (85), suggesting that in this *Tis21*-dependent mechanism mTOR might be more directly involved than AKT. It is possible that the cell type and/or the genetic background (i.e., *Patched1 *heterozygous) influence the inhibitory mechanism of action of *Btg2*/*Tis21* on the PI3K/AKT/mTOR pathway. Indeed, it is worth noting that in the P7 GCPs of the high frequency MB model *Ptch1^+/−^/Tis21^KO^* two activators of PI3K/AKT, i.e., *Timp1* (58) and *Sik2* (59) are down-regulated; likewise, the antagonist of mTOR *Smg1* (60) is down-regulated, while the mTOR activators *Lars*, *Rraga* and *Dgkq* (65, 66) are overexpressed. We have observed this differential regulation also in MB tumors (**Figure 3A**), and is possible that it will be detectable at protein level as well, given that differentially expressed mRNAs correlate well with their protein products (86). Of note, in addition to AKT phosphorylation, the mTOR/S6K signaling could also be responsible for the increase in cell survival observed in *Ptch1^+/−^/Tis21^KO^* MBs, by its positive impact on protein translation (87) or by direct inhibitory phosphorylation of the pro-apoptotic protein BAD (88). In nodules derived from transplanted high frequency MB tumors we observed a striking inhibition of the phosphorylation of S6, as well as of AKT and 4EBP1, by the PI3K inhibitor MEN1611, together with strong inhibition of tumor volume, cell proliferation and survival. This implicitly confirms that the PI3K/AKT/mTOR pathway is responsible for the increased mitotic activity and cell survival. Altogether, our data suggest that there is an ongoing activation of the PI3K/AKT/mTOR pathway in the *Ptch1^+/−^/Tis21^KO^* high frequency MBs.

### Resistance to MB Treatment Generated by Positive Interaction Between the Shh and mTOR Pathways

Interestingly, in support of this possibility is the finding that there is crosstalk between the Shh and the mTOR pathways. It has been demonstrated that within the GCPs, during cerebellar development, Shh signaling differently regulates the downstream effectors of the mTOR pathway, thus promoting protein translation and increasing cell proliferation (89, 90); in turn, in tumor cells the mTOR/S6K1 pathway can directly activate Gli1, independently of the Shh/SMO pathway, which is also a cause of resistance of tumor cells to inhibitors targeting Shh/SMO signaling (91). Furthermore, it has been observed that the PI3K/AKT/mTOR pathway is mutated in more than 5% of Shh-type MBs (which could be the reason for activation of Gli1 in itself, as mentioned above), and in 12% of cases of a large cohort of human medulloblastomas (*n* = 155) this pathway was found to be activated, as judged by phospho-AKT and phospho-S6 expression; moreover, this activation was detected mainly in adult patients and was associated to a poor outcome (41). This would suggest that our high frequency MB mouse model *Ptch1^+/−^/Tis21^KO^*, carrying activation of both the Shh and the PI3K/AKT/mTOR pathways, could be a model representative of such a cohort of patients with high-risk Shh-type medulloblastoma.

### Wide Action of MEN1611 Also on Cancer Stem Cells; Possibility of Combined Cxcl3/MEN1611 Therapy

Inhibition of PI3K/AKT/mTOR signaling in cancer represents a promising therapeutic strategy, with isoform-selective PI3Kα inhibitors showing greater efficiency and less toxicity than pan-PI3K inhibitors (92). Currently, several PI3K inhibitors – e.g., BKM120 and Alpelisib – have been tested on MB cell lines for their effects, which were tumor growth inhibition and increase of apoptosis (79, 93, 94). Here, we have investigated for the first time the preclinical activity in a brain tumor of a novel, orally bioavailable PI3Kα inhibitor, MEN1611, finding a strong anti-cancer effectiveness against *Ptch1^+/−^/Tis21^KO^* MBs. Our finding of an involvement of Cxcl3 in MB development could also suggest the possibility of a combined therapy with Cxcl3 and this PI3K inhibitor. On the other hand, combination therapies have been proposed for the inhibitors against mTOR/S6K1 and Hedgehog pathways together, as possibly more effective in cancer targeting (41, 95). However, the mTOR inhibitors can activate AKT by interrupting a negative feedback regulatory loop, resulting in their anti-tumor activity attenuated in patients (96). Conversely, our data indicate that MEN1611 causes the inhibition of PI3K/AKT/mTOR signaling by both blocking the activation of AKT possibly induced by mTOR inhibition and suppressing the phosphorylation of downstream targets of mTOR such as S6 and 4EBP1. Therefore, MEN1611 could increase the anti-neoplastic effects of an mTOR inhibitor, such as rapamycin, being suitable for a combined therapy. Furthermore, judging by the steady inhibition of the MB tumor growth curve, in our MB allograft model the treatment with MEN1611 does not appear to lead to drug resistance, as observed with other PI3Kα selective inhibitors in several cancer studies (97). Interestingly, MEN1611 increases apoptosis in *Ptch1^+/−^/Tis21^KO^* nodules, not only in the whole population of tumor cells, but also in the tumor stem cell CD15^+^ population, with consequent decrease of stem cell number. This suggests that this PI3K inhibitor facilitates, possibly by inhibiting the AKT-Bcl2 pro-survival pathway, the death of the tumor stem cells, which are at the origin of relapses also after years. This is important considering that the Shh-type MB patients treated with Shh/SMO antagonists have rapid tumor recurrence (98, 99). Further analyses are required, also to test the possibility that MEN1611 crosses the blood-brain barrier. At any rate, different delivery routes to central nervous system may be available (e.g., intrathecal or by liposomes) (100).

All this raises a question about the physiopathological involvement of *Btg2* in human medulloblastoma tumorigenesis; indeed, we have previously observed that the expression of *Btg2* varies considerably among different types of human MB tumors, with a prevalent decrease below the level measured in control cerebellum tissue (28). This is also consistent with the large *Btg2* deregulation observed in human MBs in ONCOMINE database^6^, where in classic and desmoplastic medulloblastoma samples there is large decrease or increase (from 0.5 to 5.5 log_2_ median intensity) of *Btg2* expression, relative to control cerebellum samples (average 3.5 log_2_ median intensity), thus indicating ample deregulation of expression. Therefore, *Btg2* could be implicated as a deregulated gene in the onset of the human medulloblastoma.

## Conclusion

In conclusion, although our preclinical study in mice does not allow us to predict with certainty if MEN1611 will be effective in human MB therapy, our data are highly encouraging. Indeed, the favorable toxicity profile and the potent anti-neoplastic effects observed in mice treated with MEN1611 suggest that this PI3K inhibitor may be suitable alone or in combination with other targeted therapies for the treatment of the cohort of patients with Shh-type MBs and with down-regulation of the *Btg2* gene. Importantly, the value of the MEN1611 therapy may be general for the treatment of brain tumors and not be limited to Shh-type MBs alone, because selective inhibition of the PI3K/AKT/mTOR pathway appears to be a promising strategy also in Wnt-type MBs (101), in Group 3 MBs (102, 103) and in gliomas (104). Therefore, MEN1611 could have future relevance for the treatment of patients with different intracranial tumors. Furthermore, our high frequency MB mouse model carrying activation of both the Shh and the PI3K/AKT/mTOR pathways could be a tool suitable to represent the subset of patients with this type of medulloblastoma.

## Data Availability Statement

The gene array expression data are shown as heat map in published reference (27) and as supplementary table of reference (27) at http://www.inmm.cnr.it/tirone/. The whole microarray datasets are deposited at the Gene Expression Omnibus (GEO) repository with Accession Numbers GSE178122 and GSE178124 (https://www.ncbi.nlm.nih.gov/geo/).

## Ethics Statement

The animal study was reviewed and approved by Italian Ministry of Health (Authorizations N. 206/2017-PR and N. 872/2015-PR).

## Author Contributions

MC and FT conceived and designed this study. MC, GD’A, LM, and GP performed the experiments. MC, GD’A, LM, and FT analyzed and interpreted the data. GG and SC performed in-depth bioinformatic analysis using previously available microarray data. MC and FT wrote the manuscript. GM provided reagents and performed early experiments with MEN1611. All authors contributed to the article and approved the submitted version.

## Funding

This work was supported by Regione Lazio, POR FESR 2014-2020 Bando “Life 2020_Progetti integrati” for the project “PISTA (PI3K for Solid Tumor therApy)”; CUP F57H18000070007. This study was also supported by grants from Lazio Innova (number 85-2017-14785) and from Fondazione Giovanni Celeghin to FT. MC was recipient of a postdoctoral fellowship from the Fondazione Giovanni Celeghin and from Lazio Innova grants. GD’A was recipient of a fellowship from the Fondazione Adriano Buzzati-Traverso (Arturo Falaschi fellowship) and from Lazio Innova grant.

## Conflict of Interest

Author GM was employed by Menarini Ricerche S.p.A.

IBBC-CNR has participated actively in the project PISTA (PI3K for Solid Tumor therApy), led by Menarini Ricerche S.p.A., whose goal has been to study the anti-cancer effect of the molecule MEN1611, being Menarini group the exclusive license owner on MEN1611.

PISTA (CUP F57H18000070007) has been partially funded with a non-refundable grant by Regione Lazio (Lazio Innova) under the EU programme ERDF ROP 2014-2020 and encompasses the activities mentioned in this paper, including FT’s and GM’s personnel cost and laboratory expenses.

The remaining authors declare that the research was conducted in the absence of any commercial or financial relationships that could be construed as a potential conflict of interest.

## Publisher’s Note

All claims expressed in this article are solely those of the authors and do not necessarily represent those of their affiliated organizations, or those of the publisher, the editors and the reviewers. Any product that may be evaluated in this article, or claim that may be made by its manufacturer, is not guaranteed or endorsed by the publisher.

## Footnotes

1 The Gene Ontology Resource. http://www.geneontology.org/ [Accessed September 10, 2020].

2 Menarini. https://www.menarini.com/Home/Innovation-Research/MEN1611 [Accessed October 28, 2020].

3 Primer-BLAST NCBI. nih.gov/tools/primer-blast [Accessed October 2, 2019].

4 GSEA. https://www.gsea-msigdb.org/ [Accessed May 31, 2021].

5 STRING. https://string-db.org/ [Accessed June 1, 2021].

6 ONCOMINE. http://www.oncomine.org/ [Accessed May 6, 2020].

## Supplementary Material

Supplementary Table 1 Enrichment analysis for Gene Ontology Biological Processes related to the PI3K/AKT/mTOR pathway. The 13 genes deregulated in the pairwise comparison *Ptch1^+/−^/Tis21^KO^* *versus* *Ptch1^+/−^/Tis21^WT^* belonging to the PI3K/AKT/mTOR pathway (see **Figures 1** and **2**) were used to perform an enrichment analysis for Gene Ontology (GO) Biological Processes by the Panther Classification System. The raw *p* values were determined by Fisher’s exact test and were corrected with the False Discovery Rate (FDR) calculated by the Benjamini-Hochberg procedure. The genes listed in **Figure 2** resulted overrepresented in the following GO Biological Processes: regulation of mTOR signaling (GO:0032006) with a FDR value of 0.00275; regulation of intracellular signal transduction (GO:1902531) with a FDR value of 0.00292; regulation of mTORC1 signaling (GO:1903432) with a FDR value of 0.00485.

Supplementary Figure 1 A protein-protein interaction analysis by the STRING software, performed on the whole set of genes significantly deregulated in *Ptch1^+/−^/Tis21^KO^* relative to the *Ptch1^+/−^/Tis21^WT^* GCPs, showed the interaction networks of the subset of 13 deregulated genes expanded to no more than their ten 1^st^ shell interactors. Among the enriched GO biological processes, two are particularly relevant to our model and are highlighted in **Figure S1** with different colors: red, GO:0006413 translational initiation (FDR 0.00083); purple, GO:0032006 regulation of TOR signaling (FDR 0.0442).

## Abbreviations

ANOVA, analysis of variance; CD15, cluster of differentiation 15; EGL, external granule layer; FDR, Benjamini and Hochberg false discovery rate; GCPs, granule cell precursors; IGL, internal granule layer; KO, knockout; MB, medulloblastoma; mTOR, mammalian target of rapamycin; P7, postnatal day 7; PBS, phosphate buffered saline; PFA, paraformaldehyde; PI3K, phosphatidylinositol-3-kinase; Ptch1, Patched1 gene; SEM, standard error of the mean; Shh, Sonic hedgehog; WT, wild-type.
