# Supplementary figures and images for: Tumor Growth in the High Frequency Medulloblastoma Mouse Model Ptch1+/−/Tis21KO Has a Specific Activation Signature of the PI3K/AKT/mTOR Pathway and Is Counteracted by the PI3K Inhibitor MEN1611
Source: Front Oncol. 2021 Jul 30;11:692053. doi: 10.3389/fonc.2021.692053 (PMC8362831; doi:10.3389/fonc.2021.692053)

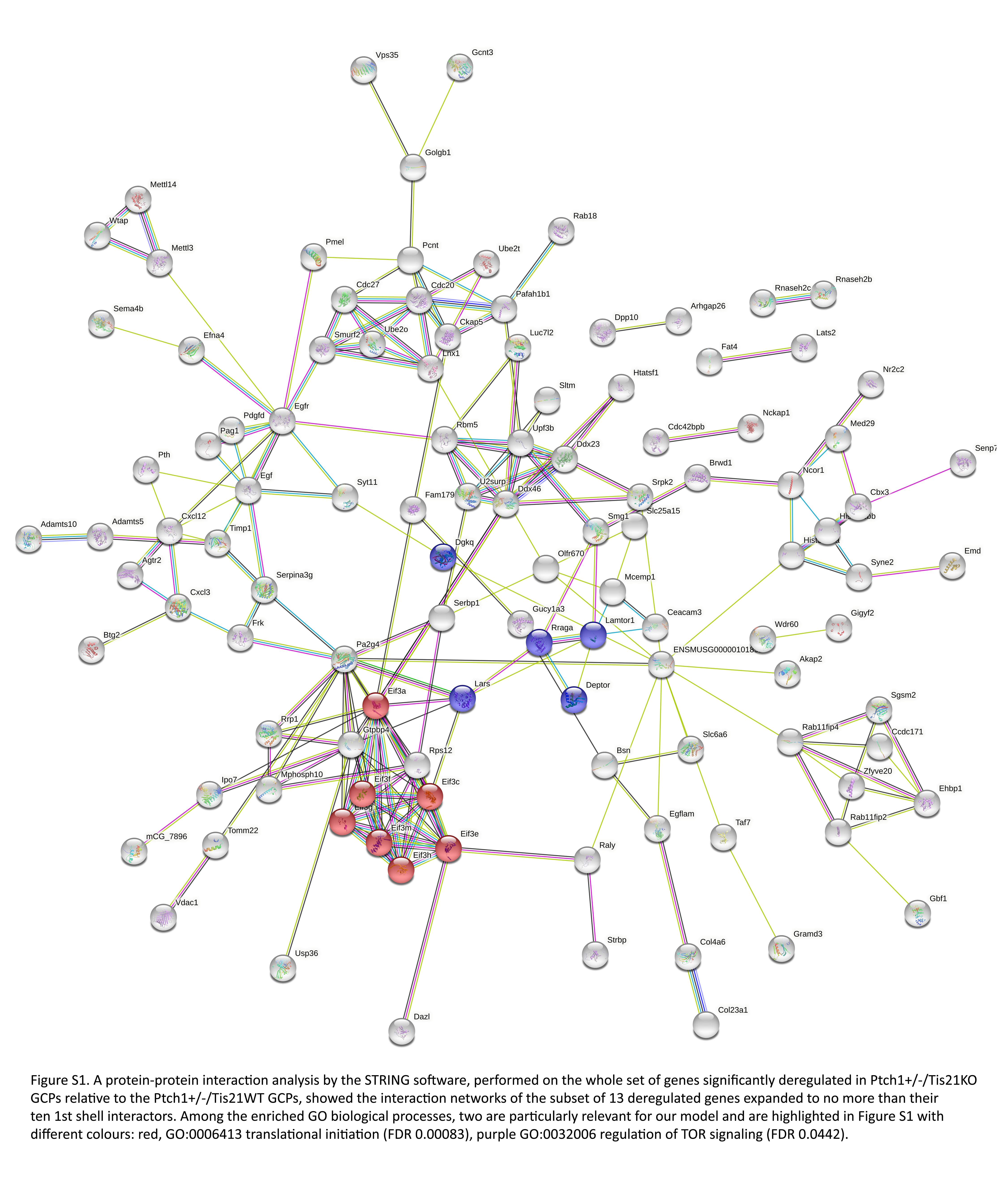

Supplement: Supplementary Figure 1 — A protein-protein interaction analysis by the STRING software, performed on the whole set of genes significantly deregulated in Ptch1+/−/Tis21KO relative to the Ptch1+/−/Tis21WT GCPs, showed the interaction networks of the subset of 13 deregulated genes expanded to no more than their ten 1st shell interactors. Among the enriched GO biological processes, two are particularly relevant to our model and are highlighted in Figure S1 with different colors: red, GO:0006413 translational initiation (FDR 0.00083); purple, GO:0032006 regulation of TOR signaling (FDR 0.0442). [file Image_1.tif]
